# Impacts of Early Weaning on Lamb Gut Health and Immune Function: Short-Term and Long-Term Effects

**DOI:** 10.3390/ani15142135

**Published:** 2025-07-18

**Authors:** Chong Li, Yunfei Xu, Jiale Jia, Xiuxiu Weng, Yang Zhang, Jialin Peng, Xueming An, Guoxiu Wang

**Affiliations:** 1 College of Animal Science and Technology, Gansu Agricultural University, Lanzhou 730070, China; lichong@gsau.edu.cn (C.L.); 18893987642@163.com (Y.X.); jiajiale0927@163.com (J.J.); zy1802907433@163.com (Y.Z.); 2 The State Key Laboratory of Grassland Agro-Ecosystems, College of Pastoral Agriculture Science and Technology, Lanzhou University, Lanzhou 730020, China; wengxx@lzu.edu.cn; 3 Linxia Animal Husbandry Development Center, Linxia 731800, China; pjl8356@163.com (J.P.); anxueming369@163.com (X.A.)

**Keywords:** early weaning, intestines, transcriptomics, oxidative stress, immunity

## Abstract

Despite the known impacts of weaning on animal health, the underlying molecular mechanisms remain unclear, particularly how psychological and nutritional stress differentially affect gut health and immune function over time. This study hypothesized that early weaning exerts distinct short- and long-term effects on lamb stress physiology, immunity, and gut health, mediated by specific molecular pathways. Twelve pairs of full-sibling male Hu sheep lambs were assigned to control (CON) or early-weaned (EW) groups. Plasma stress/immune markers were dynamically monitored, and intestinal morphology, antioxidant capacity, apoptosis, and transcriptomic profiles were analyzed at 5 and 28 days post-weaning. Early weaning triggered transient psychological stress, elevating hypothalamic–pituitary–adrenal (HPA) axis hormones (cortisol, catecholamines) and inflammatory cytokines (TNF-α) within 1 day (*p* < 0.05); however, stress responses were transient and recovered by 7 days post-weaning. Sustained intestinal remodeling was observed in EW lambs, featuring reduced ileal villus height, increased crypt depth (*p* < 0.05), and oxidative damage (MDA levels doubled vs. CON; *p* < 0.01). Compensatory epithelial adaptation included increased crypt depth but paradoxically reduced villus tip apoptosis. The transcriptome analysis revealed significant changes in gene expression related to immune function, fat digestion, and metabolism. Key DEGs included *APOA4*, linked to lipid transport adaptation; *NOS2*, associated with nitric oxide-mediated immune–metabolic crosstalk; and mitochondrial gene *COX1*, reflecting energy metabolism dysregulation. Protein–protein interaction analysis revealed *NOS2* as a hub gene interacting with *IDO1* and *CXCL11*, connecting oxidative stress to immune cell recruitment. Early weaning exerts minimal lasting psychological stress but drives persistent gut dysfunction through transcriptome-mediated changes in metabolic and immune pathways, highlighting key genes such as *APOA4*, *NOS2*, and *COX1* as potential regulators of these effects.

## Simple Summary

This study investigated how early weaning affects lamb health by examining stress, immunity, and gut function. Early-weaned lambs experienced short-term psychological stress, marked by increased stress hormones and inflammation, but these effects resolved within a week. However, long-term gut changes were observed, including compromised intestinal structure, increased cell damage caused by oxidation, and adaptive changes in intestinal cells. Gene analysis highlighted key molecules involved in immune function, fat metabolism, and energy regulation. The *nitric oxide synthase 2* (*NOS2*) gene may play a key role in connecting cell stress to immune reactions. Overall, early weaning caused lasting gut issues due to changes in metabolism and nutrition, while psychological stress was temporary.

## 1. Introduction

Weaning is a critical period in the growth of lambs, marking the transition from milk to solid feed. This transition involves substantial nutritional and psychological changes that can impose significant stress on the animal, potentially affecting gut health, metabolism, and immune function [1,2]. In current production systems, lambs are typically weaned at 8 to 10 weeks of age, and earlier weaning often results in more severe stress responses [2,3]. While the physiological, emotional, and behavioral impacts of weaning on young ruminants have been widely studied [4,5,6], the exact mechanisms—especially how psychological and nutritional stress influence gut health—remain poorly understood. The relative contributions of psychological versus nutritional stress in affecting gut health, as well as their short- and long-term impacts, have not been fully characterized. Additionally, the rapid turnover of gut tissues complicates our understanding of both immediate and lasting effects on gut morphology and function.

Previous studies demonstrated that early weaning induces both psychological and nutritional stress in lambs [7,8]. Psychological stress primarily arises from separation from the mother [9], activating the hypothalamic–pituitary–adrenal (HPA) axis and increasing levels of stress hormones such as cortisol (CORT) and catecholamines [10]. This activation triggers an emergency response in the body, altering heart rate, blood pressure, body temperature, muscle tension, and metabolic rate [11]. While these responses are adaptive in the short term, they may have negative consequences, particularly for young animals with developing immune systems [2]. By contrast, nutritional stress results from the abrupt shift in diet, with structural carbohydrates replacing lactose and milk fats as the primary energy source. Our previous research showed that early weaning at 21 days significantly affects nutrient intake and digestion efficiency in lambs, particularly reducing fat digestion [8]. This dietary change demands significant adaptation by the lamb’s digestive system, and alterations in nutrient intake can disrupt metabolism and other physiological processes.

Gut health is particularly sensitive to stress. During stress, the gut mucosa exhibits structural and functional disruptions, including dysregulated epithelial cell proliferation, differentiation, and apoptosis [12]. These alterations disrupt the delicate balance of intestinal homeostasis, often manifesting as villus atrophy, crypt hyperplasia, and impaired barrier function. Specifically, stress-induced hyperproliferation of crypt base cells, coupled with accelerated differentiation and reduced villus cell survival, leads to structural and functional compromise of the intestinal epithelium [13]. The disruption of gut immunity, a critical component of the overall immune system, can increase susceptibility to infections. Post-weaning diarrhea (PWD), a common issue in young animals [14], typically occurs within 3 to 10 days after weaning, and is a leading cause of morbidity and mortality [15]. PWD is also associated with reduced weight gain and long-term production performance [16]. Furthermore, weaning-induced oxidative stress, lipid peroxidation, and inflammation also exacerbate gut damage [17,18].

Despite the known impacts of weaning on animal health, the underlying molecular mechanisms remain unclear. Specifically, how psychological and nutritional stress differentially affect gut health and immune function in the short and long term is not well understood. The present study hypothesizes that early weaning has distinct short-term and long-term effects on lamb stress, immunity, and gut health, mediated by specific molecular mechanisms. Early weaning is expected to affect gut nutrient digestion and absorption, particularly the absorption of fats, by modulating genes involved in these processes. These molecular changes are anticipated to influence key metabolic and immune regulatory pathways, leading to both immediate and prolonged consequences for lamb gut health, metabolism, and immune function.

This study aimed to clarify how early weaning affects lamb health by examining its short-term and long-term impacts on stress responses, gut function, and immune regulation. Transcriptome analysis was performed to identify the molecular mechanisms linking metabolic stress and immune dysregulation to gut dysfunction. The findings enhance our understanding of the short- and long-term impacts of early weaning on lamb health and highlight potential strategies for mitigating the adverse effects of weaning on gut function and immunity.

## 2. Materials and Methods

### 2.1. Experimental Design

To minimize the impact of genetic background differences on the experimental results, 12 pairs of full-sibling neonatal male Hu lambs were selected from a commercial sheep farm. Within each pair, one lamb was assigned to the control group (CON, birth weight = 3.35 ± 0.76 kg, *n* = 12) and the other to the early weaning group (EW, birth weight = 3.26 ± 0.62 kg, *n* = 12). From birth to 6 days of age, lambs were housed indoors with their ewes to ensure sufficient colostrum intake. At 7 days of age, all lambs were separated from their dams and fed exclusively with a milk replacer (23.22% CP, 13.20% lipid) at 2% of average body weight per day (air-dry basis) following the manufacturer’s recommendations. The milk replacer was reconstituted at a concentration of 200 g/L of water and administered at 40 °C. At 21 days of age, all lambs in the EW group (body weight = 5.63 ± 0.23 kg, starter intake = 121.70 ± 14.12 g/d) were abruptly weaned, while those in the CON group (body weight = 5.64 ± 0.25 kg, starter intake = 103.44 ± 12.79 g/d) continued to receive artificial feed until 49 days of age. Abrupt weaning was adopted to reflect the common practice in large-scale, indoor sheep production systems in China, where gradual weaning is often impractical due to the large number of animals and labor constraints. This design enabled us to examine the physiological effects of early maternal separation and weaning stress by comparing early-weaned lambs at 5 days post-weaning (26 days of age) and 28 days post-weaning (49 days of age) with non-weaned lambs at the same ages, under otherwise similar feeding and management conditions. Throughout the study, all lambs had ad libitum access to water and were provided a pelletized starter diet from 7 days of age (Table 1). Diets were formulated to meet the requirements of the “Feeding standard of meat-producing sheep and goats (NY/T 816-2004)” [19] published in China.

### 2.2. Sample Collection

Blood samples were collected from all lambs via jugular venipuncture in the morning before feeding at 0 days (21 days of age), 1 day (22 days of age), 2 days (23 days of age), 3 days (24 days of age), 7 days (28 days of age), 14 days (35 days of age), 21 days (42 days of age), and 28 days (49 days of age) post-weaning. Each blood sample was collected into a 5 mL lithium heparin (LH) anticoagulant tube and a 2 mL K_2_EDTA anticoagulant tube. After collection, blood in the LH tube was centrifuged at 3000× *g* for 15 min to harvest plasma, which was stored at −20 °C for the measurement of stress-related hormones, HPT, and TNF-α. The blood in the K_2_EDTA tube was analyzed immediately for hematological parameters.

At 26 days of age (5 days post-weaning) and 49 days of age (28 days post-weaning), six lambs from each group were randomly selected and slaughtered via jugular vein exsanguination after a 12 h fasting period. The lambs were restrained to minimize stress prior to slaughter, and exsanguination was performed swiftly to ensure minimal suffering. The lambs were not anesthetized during the procedure, as the method of exsanguination was deemed humane and in accordance with the guidelines for the ethical treatment of experimental animals approved by the Gansu Agricultural University’s Academic Committee. Immediately post-slaughter, intestinal tissue samples were collected from the middle section of the duodenum, the anterior section of the jejunum (specifically from a segment located 0.5 to 1.0 m posterior to the end of the duodenum), the middle section of the ileum, and the middle section of the colon (at the center of the colonic loop). Before collecting the samples, they were thoroughly rinsed with 1× phosphate-buffered saline (PBS). Samples were fixed in 4% paraformaldehyde for histological examination. Additionally, samples from the middle section of the ileum were snap-frozen in liquid nitrogen and stored at −80 °C for total RNA extraction.

### 2.3. Measurement of Hematological Parameters

A PROKAN PE6800 Blood Analyzer (PROKAN Electronics Inc., Shenzhen, China) was used to estimate the number of total white blood cells (WBCs), neutrophils (NEUs), lymphocytes (LYMs), red blood cells (RBCs), and the concentration of hemoglobin (Hb) in K_2_EDTA anticoagulant blood samples.

### 2.4. Measurement of Plasma Stress-Related Hormones, Haptoglobin, and TNF-α

The concentrations of plasma cortisol (CORT), norepinephrine (NE), haptoglobin (HPT), and tumor necrosis factor-alpha (TNF-α) were measured using appropriate enzyme-linked immunosorbent assay (ELISA) kits (Abcam, Cambridge, UK). Determinations were performed using a microplate reader (Thermo Fisher Scientific, Vantaa, Finland).

### 2.5. Measurement of Intestinal Morphology

Duodenum, jejunum, ileum, and colon samples were fixed with 4% paraformaldehyde, embedded in paraffin, sectioned (5 μm), and stained with hematoxylin and eosin (HE). The slices were observed using an optical microscope (BA210 Digital, Motic China Group Co. Ltd., Xiamen, China), and 12 complete intestinal villi were randomly selected from each slice. The height, width, and depth of the fossa and muscularis of intestinal villi were measured using an image analysis system (Motic Image Plus 2.0, Motic China Group Co. Ltd., Xiamen, China).

### 2.6. Measurement of Apoptosis in Ileal Cells

Terminal deoxynucleotidyl transferase dUTP nick-end labeling (TUNEL) staining was performed using a TUNEL kit (Roche, Basel, Switzerland) according to the manufacturer’s instructions to assess apoptosis in intestinal tissue samples. Briefly, ileum tissue samples fixed with 4% paraformaldehyde were embedded in paraffin and sliced (5 μm). The TUNEL reaction mixture was then prepared, applied to tissue sections, and incubated at 37 °C in a humidified chamber. The stained sections were observed under a microscope (BA210 Digital, Motic China Group Co. Ltd., Xiamen, China) to detect apoptotic cells.

### 2.7. Measurement of Antioxidant and Immune Indices in Ileum

The collected intestinal tissues were ground in liquid nitrogen and suspended in pre-cooled 1 × PBS. After full homogenization, intestinal tissues were centrifuged at 3000× *g* for 10 min at 4 °C to obtain supernatants and prepare 10% tissue extracts. The activities of total superoxide dismutase (SOD) and glutathione peroxidase (GSH-Px), as well as the concentrations of malondialdehyde (MDA) and immunoglobulin A (IgA) were measured using appropriate kits (Nanjing Jiancheng Institute of Biological Engineering, Nanning, China). Determinations were performed using a Thermo Scientific™ Varioskan™ LUX multimode microplate reader (Thermo Fisher Scientific, Vantaa, Finland).

### 2.8. mRNA Library Construction and Sequencing

Total RNA was extracted from ileum samples using an RNA extraction kit (Takara, Kusatsu, Japan). The RNA integrity was analyzed by 1% agarose gel electrophoresis, and RNA purity and concentration were determined using a Nanodrop2000 instrument (Thermo Fisher Scientific, Wilmington, NC, USA). The quality and purity of total RNA were further assessed using a Bioanalyzer 2100 instrument and a LabChip kit (Agilent, Santa Clara, CA, USA), ensuring that all samples had RNA integrity number (RIN) values > 7.0. Approximately 10 μg of total RNA was used to isolate poly(A) mRNA with poly-T oligo attached magnetic beads (Invitrogen, Carlsbad, CA, USA). Following purification, mRNAs were fragmented into small pieces using divalent cations at an elevated temperature. These fragments were reverse-transcribed, and the final cDNA library was obtained using an mRNA Seq sample preparation kit (Illumina, San Diego, CA, USA) following the manufacturer’s instructions. The average insert size for paired-end libraries was 300 ± 50 bp. Subsequently, paired-end sequencing was performed on an Illumina HiSeq 4000 platform (Illumina) according to the manufacturer’s instructions.

Raw data generated by sequencing were filtered using Cutadapt to exclude unqualified sequences. Adapter reads, reads with undetermined base information within the total number of raw reads exceeding 5%, and low-quality reads (base number of mass value Q ≤ 10 accounting for > 20% of the whole read) were removed. Clean reads were then obtained by verifying sequence quality using FastQC (http://www.bioinformatics.babraham.ac.uk/projects/fastqc/, accessed on 1 May 2024), including Q20, Q30, and GC content. All downstream analyses were performed using high-quality clean data. Raw sequence data have been submitted to the NCBI Sequence Read Archive under accession code SRP567385.

Reads were aligned against the UCSC sheep reference genome (http://genome.ucsc.edu/, accessed on 1 May 2024) using the HISAT package, which initially removes a portion of reads based on their quality, then maps them to the reference genome. HISAT builds a database of potential splice junctions and confirms them by comparing previously unmapped reads against the database of putative junctions. Mapped reads for each sample were assembled using StringTie [20], and all sample transcriptomes were then merged to reconstruct a comprehensive transcriptome using Perl scripts. StringTie was employed to assess gene expression levels by calculating fragments per kilobase per million mapped fragments (FPKM) [20]. DEGs were identified using DESeq2 package in R (v4.3.1) based on |log_2_ FC| > 1 and *p* < 0.05, while false discovery rate (FDR) adjusted *Q*-values were also calculated. Functional categories of DEGs were established using the Kyoto Encyclopedia of Genes and Genomes (KEGG) database. KEGG enrichment analyses were performed using KOBAS (http://bioinfo.org/kobas/, accessed on 27 January 2025) with a hypergeometric test, and *p*-values were corrected using the False Discovery Rate (FDR). The PPI network of DEGs at 49 days of age was constructed using the STRING database (version 12.0) with a confidence score threshold of >0.15.

### 2.9. Statistical Analysis

Data were analyzed using SPSS version 22.0 (IBM Corp., Armonk, NY, USA). Two-way analysis of variance (ANOVA) within the general linear model (GLM) framework was conducted to evaluate the main effects of weaning strategy and age, as well as their interaction, on hematological parameters, plasma stress-related hormones, and intestinal morphology. Additionally, *t-*tests were performed to determine the significance of differences between EW and CON groups for each variable at the same age. Each dependent variable was tested for normality and homogeneity of variance prior to analysis. Statistical significance was set at *p* < 0.05.

## 3. Results

### 3.1. Hematological Responses

Interactions between weaning and age had no significant effect on the total WBC count, NEU count, LYM count, RBC count, and Hb concentration (*p* > 0.05; Table 2). Weaning had a significant effect on the number of WBC and LYM in lamb blood (*p* < 0.05), and age had a significant effect on the number of LYM, RBC, and Hb concentration (*p* < 0.05). The number of WBCs in the EW group was significantly higher than in the CON group at 1 day after weaning (*p* < 0.05). Similarly, the LYM count was higher at 3 days after weaning, and the NEU count was higher at 1 day after weaning (*p* < 0.05). No significant differences in hematological indicators were observed between the two groups at other time points (*p* > 0.05).

### 3.2. Plasma Stress-Related Hormones, Haptoglobin, and TNF-α

The effects of weaning and interactions between weaning and age on plasma CORT, HPT, NE, and TNF-α concentrations in lambs were not significant (*p* > 0.05; Table 3). Age had a significant effect on plasma NE concentration (*p* < 0.05). There were no significant differences between days of age for concentrations of CORT, HPT, NE, and TNF-α in the CON group (*p* > 0.05), but the concentration of NE in the EW group was higher at 2 days after weaning than at 0 and 7 days after weaning (*p* < 0.05). TNF-α was significantly higher on day 1 after weaning than on day 0 in the EW group (*p* < 0.05). CORT, HPT, and TNF-α levels in the EW group were significantly higher than in the CON group on day 1 after weaning (*p* < 0.05), and NE levels were significantly higher than in the CON group on day 2 after weaning (*p* < 0.05). There were no significant differences in other days of age between the two groups (*p* > 0.05).

### 3.3. Intestinal Morphology

Interactions between weaning and age had no significant effect on the morphology of the duodenum, jejunum, ileum, and colon of lambs (*p* > 0.05; Table 4). Weaning significantly reduced the height of ileum villi (*p* < 0.05) and increased the depth of jejunum and ileum crypts (*p* < 0.05). The height of villi in the EW group was significantly lower than in the CON group at both 5 and 28 days post-weaning (*p* < 0.05). The depth of jejunum and ileum crypts in the EW group was significantly higher than in the CON group at 5 days post-weaning (*p* < 0.05).

### 3.4. Apoptosis of Ileal Cells

**Figure 1:**
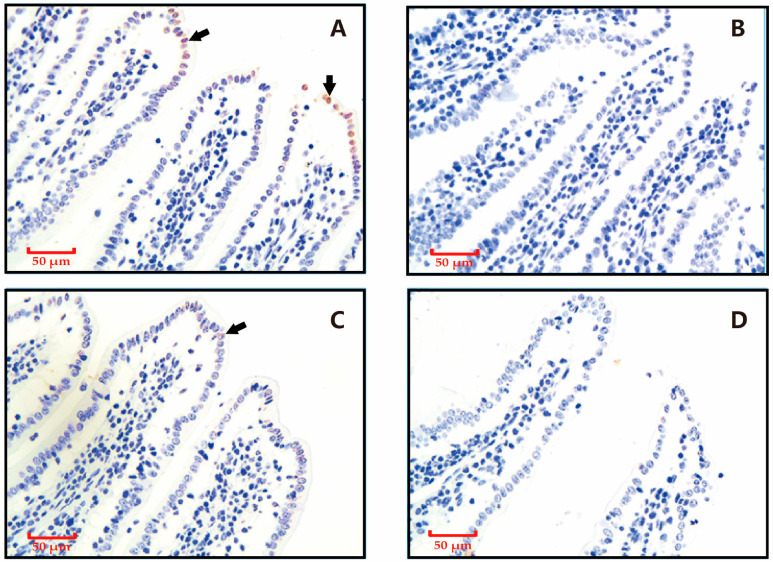
Effects of weaning on apoptotic cell distribution in ileal samples revealed by terminal deoxynucleotidyl transferase dUTP nick-end labeling (TUNEL) staining. Nuclei of apoptotic cells are brown (as indicated by the arrow), and those of non-apoptotic cells are blue. (**A**) Control group sampled at 28 days post-weaning. (**B**) Weaning group sampled at 28 days post-weaning. (**C**) Control group sampled at 5 days post-weaning. (**D**) Weaning group sampled at 5 days post-weaning.

As shown in Figure 1, the TUNEL staining revealed that apoptotic cells in ileum tissues were predominantly localized to the epithelial cells at the villus tips, characterized by distinct brown nuclear staining. Weaning reduced the proportion of TUNEL staining-positive intestinal villi and the proportion of apoptotic cells in TUNEL staining-positive intestinal villi epithelial cells.

### 3.5. Antioxidant and Immune Indices in Ileum

Interactions between weaning and age had no significant effect on the activities of SOD and GSH-Px and the levels of MDA and IgA in the ileum of lambs (*p* > 0.05; Table 5). The MDA concentration increased significantly with increasing days of age (*p* < 0.05). Weaning significantly reduced the concentration of IgA (*p* < 0.05) and increased the concentration of MDA (*p* < 0.1). IgA concentration in the EW group at both 5 and 28 days post-weaning was significantly lower than in the CON group (*p* < 0.05), while the MDA concentration was significantly higher at 28 days post-weaning (*p* < 0.05).

### 3.6. RNA Sequencing (RNA-Seq) Data Mapping and Annotation

A total of 24 cDNA libraries were sequenced from the ileum tissues of all experimental lambs (*n* = 6 per group). After removing adaptors and filtering, we obtained 1187.7 M valid reads. After mapping clean reads to the ovine genome, 87.70–89.47% of reads were successfully aligned, and 55.30–66.92% of reads had unique genomic locations. Moreover, 80.31–83.44% of reads were paired end-mapped reads (Supplementary Material Table S1).

### 3.7. Differentially Expressed Genes (DEGs)

**Figure 2:**
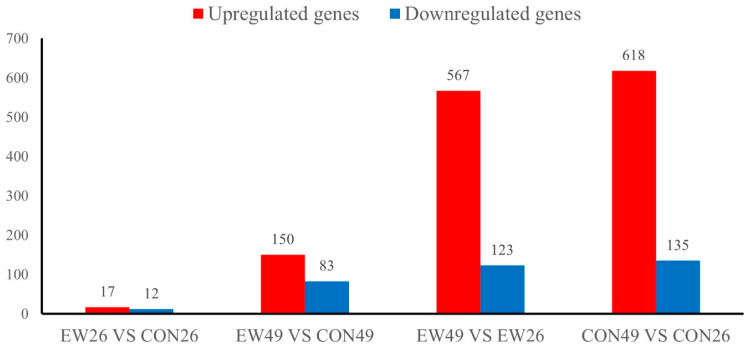
Number of differentially expressed genes (DEGs). CON26 and CON49 refer to unweaned control lambs sampled at 26 and 49 days of age, corresponding to 5 and 28 days post-weaning in the weaned groups. EW26 and EW49 refer to lambs weaned at 21 days of age and sampled at 5 and 28 days post-weaning, respectively.

In the RNA-seq analysis, 18,716 genes were detected in the jejunum of all 24 individuals. Comparing the gene expression profiles between unweaned lambs at day 49 of age (CON49, corresponding to 28 days post-weaning) and lambs weaned at 21 days and sampled at day 49 (EW49, 28 days post-weaning), we identified 233 differentially expressed genes (DEGs; Figure 2). Among these, 83 were downregulated and 150 upregulated. The comparison between weaned lambs sampled at day 26 of age (EW26, 5 days post-weaning) and age-matched unweaned controls (CON26, day 26 of age, 5 days post-weaning equivalent) detected 29 DEGs, of which 17 were upregulated and 12 were downregulated. Comparing EW49 and EW26 groups detected 690 DEGs, among which 567 were upregulated and 123 were downregulated. Comparing CON49 and CON26 groups, we detected 753 DEGs, among which 618 were upregulated and 135 were downregulated.

DEGs between CON49 and EW49, and CON26 and EW26, are listed in the Supplementary Material Table S2. Table 6 and Table 7 list the top 20 DEGs in the EW groups compared with the CON groups at 28 and 5 days post-weaning, respectively.

### 3.8. Correlation Between the Top 20 DEGs and Ileum Morphology and Antioxidant Indices

**Figure 3:**
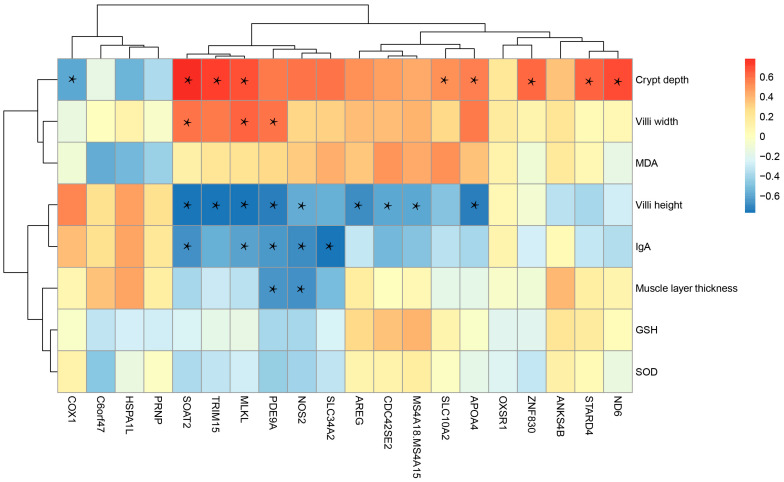
Correlation between the top 20 DEGs and ileum morphology and antioxidant indices at 28 days post-weaning. * Indicates a statistically significant correlation (*p* < 0.05, |r| > 0.5).

Spearman’s correlation analysis was performed between the top 20 DEGs and ileum morphology and antioxidant indices at 28 days post-weaning (Figure 3). The results showed that among the 20 most significantly differentially expressed genes, villus height in the ileum was significantly correlated (*p* < 0.05, |r| > 0.5) with the expression levels of nine genes, including *APOA4*, *SOAT2*, and *MLKL*. Crypt depth was significantly correlated with the expression levels of nine genes, including SOAT2, ND6, and TRIM15 (*p* < 0.05, |r| > 0.5). Villus width was significantly associated with *SOAT2*, *ND6*, and *PDE9A* (*p* < 0.05, |r| > 0.5). Muscularis thickness showed significant correlations with *NOS2* and *PDE9A* (*p* < 0.05, |r| > 0.5). In addition, the IgA concentration was significantly correlated with five genes, including NOS2 (*p* < 0.05, |r| > 0.5).

### 3.9. KEGG Pathway Analysis of DEG

**Figure 4:**
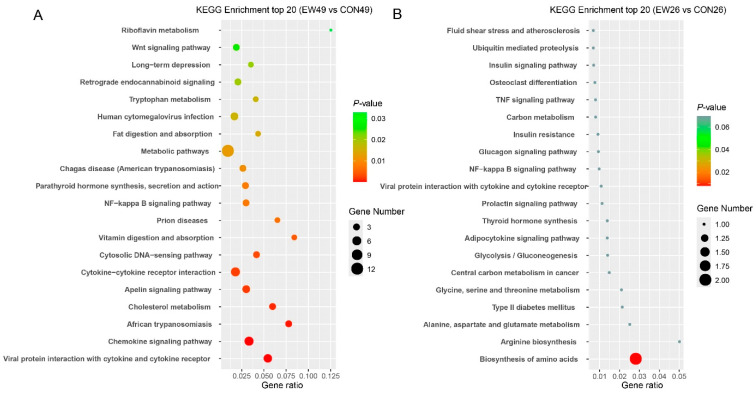
Results of KEGG enrichment analysis. (**A**) EW49 vs. CON49. (**B**) EW26 vs. CON26. The x-axis represents enrichment scores, and the y-axis represents pathway terms. Circle color indicates false discovery rate (FDR) based on *p*-value, and circle size indicates the number of DEGs. CON26 and CON49 refer to unweaned control lambs sampled at 26 and 49 days of age, corresponding to 5 and 28 days post-weaning in the weaned groups. EW26 and EW49 refer to lambs weaned at 21 days of age and sampled at 5 and 28 days post-weaning, respectively.

To explore the biological functions of the identified DEGs, a KEGG enrichment analysis was performed (Figure 4). At 28 days post-weaning, there were 21 significantly enriched pathways (*p* < 0.05) in the EW group compared with the CON group. The most notable pathways were primarily associated with immune-related processes, including viral protein interactions with cytokine and cytokine receptors, chemokine signaling pathways, and cytokine–cytokine receptor interactions. Additionally, pathways related to lipid absorption and metabolism, such as cholesterol metabolism and fat digestion and absorption, were significantly enriched. By contrast, at 5 days post-weaning, only a single pathway, biosynthesis of amino acids, was significantly enriched (*p* < 0.05) in the EW group compared to the CON group.

### 3.10. Protein–Protein Interaction (PPI) Network of DEGs

The PPI network of DEGs at 28 days post-weaning was constructed using the STRING database (version 12.0) with a confidence score threshold of >0.15. The resulting network includes 160 nodes and 109 edges, with an average node degree of 1.36 (Figure 4).

**Figure 5:**
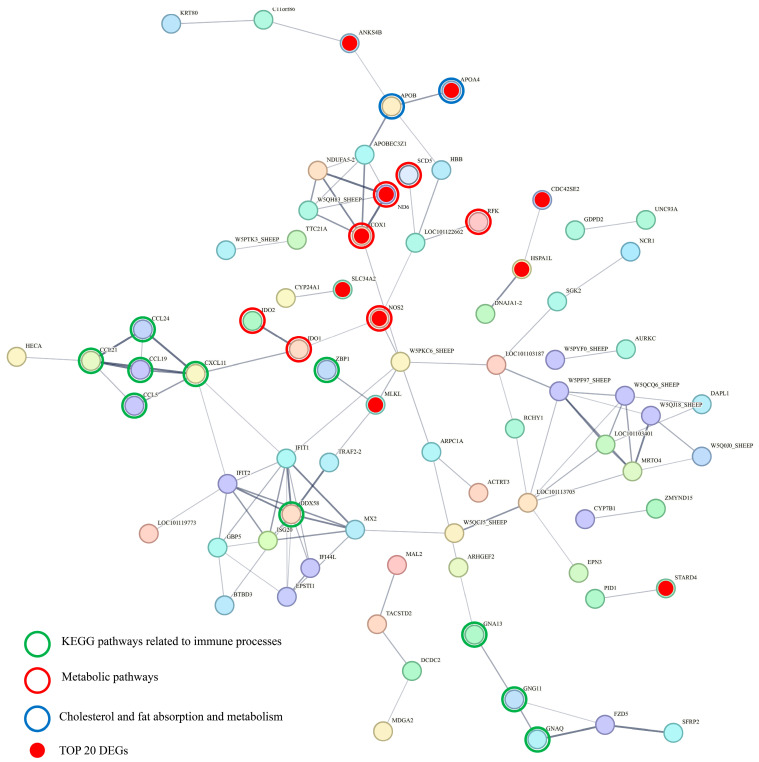
Protein–protein interaction network of DEGs at 49 days of age.

Further integration of PPI network analysis with KEGG pathway enrichment analysis revealed significant interactions among the most significantly enriched pathways, particularly those related to immune processes, metabolic pathways, and lipid absorption and metabolism. Notably, the top 20 most significant DEGs were highly concentrated within the PPI network, with prominent associations with KEGG pathways related to metabolic processes and lipid metabolism, including cholesterol and lipid absorption and metabolism. Key genes, including cytochrome c oxidase subunit 1 (*COX1*), nitric oxide synthase 2 (*NOS2*), indoleamine 2,3-dioxygenase 1 (*IDO1*), and C-X-C motif chemokine ligand 11 (*CXCL11*), were identified as pivotal nodes, linking these enriched pathways and highlighting their central roles in immune-related processes and metabolic regulation (Figure 5).

## 4. Discussion

Our previous research showed that early-weaned lambs (weaned at 21 days) had lower growth performance by 28 days post-weaning compared with lambs that remained unweaned during the same period, with body weights of 9.74 kg versus 12.88 kg and average daily gains of 146.79 g/d versus 258.57 g/d in unweaned lambs [8]. Despite increased solid feed intake, early weaning reduced nutrient utilization, evidenced by lower crude protein and fat digestibility [8]. Our previous study found that daily fat digestion per kg BW was significantly lower in early-weaned lambs (0.40 g/kg BW) compared to unweaned lambs (2.65 g/kg BW) [8]. This represented a 6.6-fold reduction in fat digestion capacity and highlighted the profound metabolic challenges imposed by the abrupt dietary transition. Weaning stress impacts lambs in two major ways: psychologically and nutritionally [9]. Regarding psychological impacts, separation from ewes and the weaning process exert substantial influences on lambs [7]. The HPA axis is the primary regulatory system responding to stress [21]. When an individual experiences stress or threat, the HPA axis is activated, triggering a series of endocrine responses. The activation of the HPA axis puts the body into a state of heightened alert, causing significant changes in heart rate, blood pressure, body temperature, muscle tension, and metabolic levels [22]. Studies have shown that weaning stress significantly increases serum CORT levels in calves and lambs [2,10,23]. Additionally, the immature adaptive immune system of young animals relies heavily on the HPA axis as the main regulator of the immune system, providing homeostatic feedback through glucocorticoids and influencing immune responses [24]. In weaned calves treated with lipopolysaccharide, there are significant changes in blood leukocyte distribution, cytokines, and acute phase proteins [25,26], with neutrophils serving as biomarkers for weaning stress [27,28]. Since lymphocytes are closely related to adaptive immune function [29], our results suggest that immune function, especially adaptive immunity, is still developing up to 49 days of age. Although nutrient intake is known to be crucial in regulating immune system function, with both deficiency and excess negatively affecting immunity and pathogen susceptibility [4,30], some studies indicate that nutrition has limited effects on immune responses in weaned lambs [5,31]. Our results showed that acute stress responses in lambs following weaning lasted only 1 to 3 days, with stress biomarkers such as CORT, catecholamines, and TNF-α returning to normal levels after 3 days, suggesting that short-term psychological stress does not have long-lasting effects on blood immune indicators or hormone levels.

Regarding nutritional impacts, our prior research [8] demonstrated that in early weaning, while rapidly increasing solid feed intake, the abrupt cessation of liquid milk consumption leads to reduced digestibility and total digestion of crude protein and fat, leading to a shift in energy metabolism in lambs. These nutritional changes, combined with the psychological stress of weaning, may disrupt gut metabolic functions and ultimately impair gut health. This is supported by our intestinal morphology results, which showed that early weaning led to reduced villus height and increased crypt depth, indicating structural damage to the intestinal mucosa. Numerous studies demonstrated that weaning stress leads to intestinal mucosal damage in mammals, manifested as reduced villus height and increased crypt depth [32,33]. The epithelial layer covering the small intestine forms the physical barrier of the gut and is among the most rapidly renewing tissue structures in mammals, with a cell life cycle of only 4−5 days [34]. Rapid and continuous renewal of intestinal epithelial cells depends on Lgr5+-labeled crypt base columnar cells within crypts, which proliferate and differentiate into transit-amplifying cells and migrate upwards [35]. Intestinal stem cells located at the base of crypts constantly proliferate and differentiate, moving out of crypts and towards villi to replace damaged epithelial cells. Deepened crypts indicate frequent proliferation and differentiation of intestinal stem cells, which is also a marker of epithelial cell damage [36]. Research has also shown that weaning stress impairs tight junctions between epithelial cells and increases mucosal permeability, allowing bacteria, toxins, and allergens to more easily cross the gut barrier, leading to inflammation or immune reactions [32,37]. These changes are accompanied by increased epithelial cell apoptosis, observable by TUNEL staining, and the process is potentially driven by metabolic and oxidative stress.

These findings highlight the impact of early weaning on gut health, particularly regarding mucosal integrity and cell renewal. Although psychological stress associated with weaning may not have long-lasting effects on hormones and blood immune markers, direct structural changes in the gut suggest that the early life stage is crucial, during which the lambs are more susceptible to gastrointestinal challenges [38], and nutritional changes brought on by early weaning can have significant effects. Nutritional stress resulting from the abrupt transition to solid feed likely plays an important role in disrupting gut homeostasis [39]. Further research is needed to explore the mechanisms underlying the impact of weaning on gut health.

In this study, the MDA concentration in ileal tissue of the weaned group at 49 days of age was twice that of the non-weaned group, indicating that weaning caused oxidative damage to ileal cells, which may mediate the impact of weaning on gut health. MDA is a product of lipid peroxidation and is an important marker of oxidative damage in cells. Reactions involving oxygen radicals and lipid peroxidation play a significant role in metabolism [40]. It is generally believed that the generation of reactive oxygen species (ROS) and the body’s ability to clear oxygen radicals are coordinated and in dynamic balance. When this balance is disrupted, lipid peroxidation occurs, leading to MDA production, which alters the fluidity and permeability of cell membranes, ultimately causing changes in cell structure and function [41,42]. The degree of lipid peroxidation in villus cells is significantly higher than in the crypt region, and the villus region generates numerous free radicals, which may contribute to epithelial cell damage and promote epithelial cell differentiation and migration [43]. Studies have shown that weaning induces oxidative stress in piglets [44], characterized by an increased ROS production, decreased antioxidant capacity, reduced villus height, deepened crypts, and decreased digestive enzyme activity [45]. Early weaning may inhibit the p65 and Nrf2 signaling pathways, affecting the expression of antioxidant genes and antioxidant system development [46].

Oxidative stress caused by weaning may result from significant changes in lamb food structure post-weaning, leading to an increased basal metabolic rate in the gut and consequent generation of ROS. Our previous research demonstrated that after weaning, solid feed intake rapidly increases, while both crude protein and crude fat digestion decrease, with blood biochemical indicators suggesting reduced nitrogen deposition [8]. Studies have shown that sudden reductions in nutrient bioavailability can trigger stress responses in the gut, as nutrient-rich diets typically promote effective mRNA responses and strong immune reactivity to dietary antigens and microbial metabolites [39]. Our transcriptome analysis results indicate that among the most significant DEGs, *NOS2*, *OXSR1*, *HSPA1L*, and *ND6* are all related to oxidative stress and ROS production, further confirming that weaning leads to oxidative damage in intestinal tissues.

Transcriptome analysis also provided insights into the underlying molecular mechanisms. At 49 days of age (28 days post-weaning), 320 DEGs were identified, compared to just 29 DEGs at 26 days of age (5 days post-weaning). This indicates that the effects of weaning on gene expression are not immediate but rather evolve over time. KEGG enrichment analysis indicated that at 28 days post-weaning, DEGs between weaned and control groups were mainly enriched in pathways related to fat digestion and absorption, cytokine–cytokine receptor interaction, tight junctions, chemokine signaling pathways, and cholesterol metabolism. Our previous research showed that weaning reduces fat digestibility in lambs [8], and enrichment of DEGs in these intestinal pathways confirms this effect. Additionally, the DEGs suggest that the impact of weaning on fat digestion is associated with the expression of apolipoproteins such as apolipoprotein A-IV (APOA4). Notably, APOA4 emerged as the most significantly upregulated gene, which is involved in lipid absorption, transport, and metabolism [47]. Its upregulation suggests that lambs are compensating for decreased fat intake and digestibility following weaning. Weaning is known to affect the gut fat metabolism pathway and apolipoprotein expression in lambs [33]. Furthermore, the enriched pathways indicate that the effects of weaning on transcriptional regulation in the gut are mainly related to immune function regulation and gut barrier integrity.

The DEGs in immune and gut barrier-related pathways include various interleukins, TNF-α superfamily members, and tight junction proteins. Other studies have shown that weaning upregulates immune function-related pathways in calves, and DEGs may be associated with the recovery of gut mucosal immune responses and reduced mucosal thickness during the weaning transition [48], consistent with our findings. This underscores the significant impact of increased immune responses and gut barrier damage on gut health in lambs post-weaning.

Integrating the PPI network and KEGG pathway enrichment results provides insights into the molecular mechanisms underlying the observed biological processes and their connections to phenotypic changes. The most significantly enriched KEGG pathways, including those related to immune processes, metabolic pathways, and lipid absorption and metabolism, were well represented within the PPI network. Interestingly, the top 20 most significant DEGs were clustered tightly in the network, particularly for pathways associated with metabolic processes and lipid metabolism. Among these, *APOA4* emerged as the most significantly upregulated gene. As a key player in lipid metabolism [47], its upregulation may represent a compensatory response to the reduced fat intake and digestibility observed after weaning. Other genes involved in oxidative stress and immune regulation, such as *NOS2*, *OXSR1*, *ND6*, and *COX1*, were enriched in metabolic pathways, indicating altered energy metabolism and mitochondrial function, both critical for maintaining gut health and immune responses. In addition, the correlation analysis revealed that multiple DEGs were significantly associated with ileal morphological parameters and IgA levels, suggesting their potential roles in shaping gut structural integrity and mucosal immunity.

In particular, *NOS2*, involved in nitric oxide synthesis [49], interacts with *IDO1*, a key regulator of tryptophan metabolism, which in turn interacts with *CXCL11*, forming a cascade of molecular interactions connecting metabolic regulation with immune responses. *IDO1*, a key regulator of tryptophan metabolism, modulates immune responses by influencing the balance between pro-inflammatory and anti-inflammatory pathways [50]. *CXCL11*, a chemokine involved in recruiting immune cells [51], implies the activation of immune pathways in response to metabolic stress. These interactions, together with the immune-related pathways enriched in the PPI network, highlight the connection between metabolic stress and immune function. The activation of inflammatory pathways, driven by these genes, likely contributes to increased gut permeability, further exacerbating damage to the gut epithelium.

## 5. Conclusions

While short-term psychological stress had minimal long-term effects, nutritional stress led to longer-lasting nutritional stress that harms gut structure and causes oxidative damage. Transcriptome analysis revealed that weaning altered gene expression in pathways related to immune function, fat digestion, and metabolism. Key genes like *NOS2* link metabolic changes to immune responses. Future research should focus on validating the molecular mechanisms identified and developing strategies to mitigate the adverse effects of weaning stress.

## Figures and Tables

**Table 1:** Ingredient and chemical composition of starter diet and milk replacer (air-dry basis).

Items	Starter (%)	Milk Replacer (%)
Ingredients		
Alfalfa hay	18.50	
Corn	21.00	
Extruded corn	22.30	
Soybean meal	21.50	
Extruded soybean	4.00	
Corn gluten meal	5.00	
Bran	6.00	
Limestone	0.30	
Premix *	1.00	
NaCl	0.40	
Total	100	
Chemical composition ^#^		
Dry matter	93.86	96.91
	19.50	23.22
Fat	1.33	13.20
Neutral detergent fiber	18.94	0.00
Acid detergent fiber	8.60	0.00
Starch	33.10	0.00

Notes: * The premix provided per kilogram of diet contains the following: 25 mg Fe as FeSO_4_·H_2_O; 40 mg Zn as ZnSO_4_·H_2_O; 8 mg Cu as CuSO_4_·5H_2_O; 40 mg Mn as MnSO_4_·H_2_O; 0.3 mg I as KI; 0.2 mg Se as Na_2_SeO_3_; 0.1 mg Co as CoCl_2_; 940 IU vitamin A; 111 IU vitamin D; 20 IU vitamin E; and 0.02 mg vitamin B_12_. ^#^ Dry matter, crude protein, fat, neutral detergent fiber, acid detergent fiber, and starch content were measured according to standard procedures.

**Table 2:** Effect of weaning and age on hematological responses.

Items	Treatment	Days Post-Weaning (d)							SEM	*p* Value		
		0	1	2	3	7	14	28		Weaning	Age	Weaning × Age
WBC (×10^9^ cells/L)	CON	8.29	8.59	9.03	8.31	9.20	8.28	9.86	0.223	0.024	0.328	0.464
	EW	7.95 ^b^	10.92 ^a^*	10.15 ^ab^	10.66 ^a^*	9.79 ^ab^	9.53 ^ab^	9.68 ^ab^				
LYM (×10^9^ cells/L)	CON	3.52 ^b^	3.64 ^b^	3.62 ^b^	3.32 ^b^	4.01 ^b^	4.32 ^b^	5.47 ^a^	0.108	0.041	0.004	0.528
	EW	3.47 ^b^	4.45 ^ab^	4.37 ^ab^	4.46 ^ab^*	4.53 ^ab^	4.69 ^ab^	5.06 ^a^				
NEU (×10^9^ cells/L)	CON	3.49	3.71	4.06	3.69	3.68	2.75	3.30	0.150	0.145	0.097	0.642
	EW	3.24 ^b^	5.18 ^a*^	4.41 ^ab^	4.69 ^ab^	3.75 ^ab^	3.35 ^b^	3.14 ^b^				
NEU/LYM	CON	1.02 ^ab^	1.04 ^ab^	1.21 ^a^	1.12 ^ab^	1.03 ^ab^	0.66 ^ab^	0.61^b^	0.043	0.945	0.006	0.858
	EW	1.00 ^ab^	1.31 ^a^	1.10 ^ab^	1.11 ^ab^	0.88 ^ab^	0.74 ^ab^	0.61 ^b^				
RBC (×10^9^ cells/L)	CON	8.47 ^b^	8.00 ^b^	8.02 ^b^	7.91 ^b^	7.94 ^b^	8.37 ^b^	9.22 ^a^	0.068	0.432	0.004	0.449
	EW	8.04 ^b^	8.49 ^ab^	8.34 ^ab^	8.23 ^ab^	8.25 ^ab^	8.25 ^ab^	9.08 ^a^				
Hb (g/L)	CON	122.64 ^ab^	113.86 ^ab^	111.83 ^ab^	109.79 ^b^	111.94 ^ab^	116.67 ^ab^	127.43 ^a^	1.066	0.586	0.024	0.293
	EW	113.08	120.45	115.50	113.29	109.93	112.25	121.50				

Note: CON: non-weaned group; EW: early-weaned group (weaned on day 21); WBC: white blood cells; LYM: lymphocytes; NEU: neutrophils; RBC: red blood cells; Hb: hemoglobin. * *p* < 0.05 indicates a significant difference between groups at the same age. ^a,b^, Different lowercase letters within a row indicate significant differences (*p* < 0.05).

**Table 3:** Effect of weaning and age on plasma stress-related hormones, haptoglobin, and TNFα.

Items (µg/mL)	Treatment	Days Post-Weaning (d)					SEM	*p* Value		
		0	1	2	3	7		Weaning	Age	Weaning × Age
CORT	CON	116.43	114.62	115.92	125.68	115.82	1.297	0.225	0.383	0.232
	EW	118.22	129.29 *	124.04	122.82	123.79				
HPT	CON	51.02	50.75	52.02	49.15	44.94	0.826	0.391	0.109	0.960
	EW	51.55	54.16 *	53.39	50.54	48.37				
NE	CON	1468.23	1488.63	1508.55	1544.86	1456.08	16.074	0.378	0.049	0.320
	EW	1417.75 ^b^	1542.85 ^ab^	1670.66 ^a^*	1555.05 ^ab^	1458.10 ^b^				
TNF-α	CON	97.46	94.74	94.24	95.93	90.75	0.999	0.454	0.202	0.382
	EW	97.37 ^ab^	103.94 ^a^*	93.15 ^b^	95.45ab	94.93 ^ab^				

Note: CON: non-weaned group; EW: early-weaned group (weaned on day 21); CORT: cortisol; HPT: haptoglobin; NE: norepinephrine; TNF-α: tumor necrosis factor-alpha. * indicates a significant difference between groups at the same age (*p* < 0.05). ^a,b^, Different lowercase letters within a row indicate significant differences (*p* < 0.05).

**Table 4:** Effect of weaning and age on the intestinal morphology of lambs.

	Items (μm)	5 Days Post-Weaning		28 Days Post-Weaning			*p* Value		
		CON	EW	CON	EW	SEM	Weaning	Age	Weaning × Age
Duodenum	Villi height	210.68	238.47	452.96	437.60	8.376	0.714	<0.001	0.211
	Villi width	75.88	81.21	173.27	155.40	3.717	0.818	<0.001	0.115
	Crypt depth	120.00	149.87 *	220.67	212.29	5.020	0.296	<0.001	0.070
	Muscle layer thickness	97.52	116.00 *	112.89	136.13 *	4.752	4.752	0.075	0.804
Jejunum	Villi height	430.20	412.50	447.90	413.95	11.172	0.269	0.675	0.722
	Villi width	93.08	101.92	114.62	127.15	3.437	0.144	0.005	0.793
	Crypt depth	126.95	195.33 *	159.18	181.18	7.699	0.012	0.567	0.156
	Muscle layer thickness	99.58	87.90	93.93	86.20	5.041	0.248	0.909	0.664
Ileum	Villi height	458.10 *	397.97	427.54 *	372.47	11.047	0.028	0.151	0.737
	Villi width	103.74	108.57	114.86	114.07	4.240	0.814	0.339	0.744
	Crypt depth	123.16	189.65 *	173.68	192.76	7.160	0.008	0.077	0.114
	Muscle layer thickness	112.20	103.55	93.86	88.27	8.142	0.306	0.116	0.926
Colon	Villi height	429.80	464.20	480.06	478.86	11.77	0.491	0.188	0.461
	Villi width	43.80	48.94	50.86	48.38	1.654	0.694	0.342	0.267
	Crypt depth	63.46	71.48	74.94	77.44	7.067	0.715	0.546	0.848
	Muscle layer thickness	174.82	187.52	202.28	189.24	10.187	0.993	0.484	0.537

Note: CON: non-weaned group; EW: early-weaned group (weaned on day 21). * indicates a significant difference between groups at the same age (*p* < 0.05).

**Table 5:** Effects of weaning and age on antioxidant and immune indices in the ileum.

Items	5 Days Post-Weaning		28 Days Post-Weaning			*p* Value		
	CON	EW	CON	EW	SEM	Weaning	Age	Weaning × Age
SOD (U/mg)	0.12	0.10	0.11	0.11	0.006	0.382	0.961	0.732
GSH-Px (U/mg)	1.23	1.23	1.12	1.18	0.043	0.763	0364	0.716
MDA (nmol/g)	8.53	8.29	10.13	22.30 *	1.735	0.098	0.036	0.089
IgA (mg/g)	1.31 *	0.85	1.18 *	0.99	0.060	0.014	0.904	0.268

Note: CON: non-weaned group; EW: early-weaned group (weaned on day 21). * indicates a significant difference between groups at the same age. SOD: superoxide dismutase; GSH-Px: glutathione peroxidase; MDA: malondialdehyde; IgA: immunoglobulin A; SEM: standard error of the mean.

**Table 6:** List of top 20 DEGs with the lowest *p*-value between groups at 28 days post-weaning.

Gene ID	Gene Symbol	Description	Group		log_2_FC	*p* Value	*Q* Value
			CON	EW			
MSTRG.7028	*APOA4*	Apolipoprotein A4	6.67	37.14	2.45	<0.001	<0.001
MSTRG.2698	*SLC10A2*	Solute carrier family 10 member 2	5.90	18.23	1.60	<0.001	<0.001
MSTRG.12128	*HSPA1L*	Heat shock 70 kda protein 1-like	0.69	<0.01	−9.98	<0.001	<0.001
MSTRG.5151	*PRNP*	Major prion protein	0.17	<0.01	−8.91	<0.001	0.010
MSTRG.9443	*OXSR1*	Serine/threonine-protein kinase OSR1	<0.01	4.77	11.29	<0.001	0.015
MSTRG.2150	*PDE9A*	Phosphodiesterase 9A	11.77	27.12	1.18	<0.001	0.015
MSTRG.2965	*NOS2*	Nitric oxide synthase 2	0.74	4.74	2.64	<0.001	0.019
MSTRG.19179	*CDC42SE2*	CDC42 small effector 2	8.52	20.11	1.21	<0.001	0.019
MSTRG.22642	*COX1*	Cytochrome c oxidase subunit 1	1195.86	6.55	−7.53	<0.001	0.020
MSTRG.12142	*C6orf47*	Uncharacterized protein c6orf47	1.04	<0.01	−10.56	<0.001	0.020
MSTRG.2916	*ZNF830*	Zinc finger protein 830	<0.01	0.64	8.68	<0.001	0.032
MSTRG.19825	*STARD4*	StAR related lipid transfer domain containing 4	0.81	2.71	1.71	<0.001	0.032
MSTRG.20048	*SLC34A2*	Solute carrier family 34 member 2	14.21	33.44	1.21	<0.001	0.032
MSTRG.12237	*TRIM15*	Tripartite motif containing 15	0.79	2.33	1.52	<0.001	0.047
MSTRG.22661	*ND6*	NADH-ubiquinone oxidoreductase chain 6	<0.01	4.74	8.59	<0.001	0.049
MSTRG.16608	*SOAT2*	Sterol O-acyltransferase 2	0.38	1.59	2.03	<0.001	0.052
MSTRG.12735	*MS4A18*	Membrane spanning 4-domains A18	1.69	5.27	1.61	<0.001	0.069
MSTRG.9443	*AREG*	Amphiregulin	1.21	0.85	−2.09	0.000	0.070
MSTRG.14252	*ANKS4B*	Ankyrin repeat and sterile alpha motif domain containing 4B	1.04	3.51	1.72	0.001	0.154
MSTRG.5689	*MLKL*	Mixed lineage kinase domain like pseudokinase	2.80	5.71	1.00	0.001	0.154

Note: CON: non-weaned group; EW: early-weaned group (weaned on day 21).

**Table 7:** List of top 20 DEGs with the lowest *p*-value between groups at 5 days post-weaning.

Gene ID	Gene Symbol	Description	Group		log_2_FC	*p* Value	*Q* Value
			CON	EW			
MSTRG.24683	*ZBTB33*	Zinc finger and BTB domain containing 33	3.31	1.7	−2.08	<0.001	0.922
MSTRG.16036	*RAB11FIP1*	RAB11 family interacting protein 1	2.99	1.17	−1.66	<0.001	0.922
MSTRG.21722	*AGGF1*	Angiogenic factor with G-patch and FHA domains 1	1.57	5.03	1.58	0.001	0.922
MSTRG.4224	*GNA13*	G protein subunit alpha 13	50.13	31.05	−4.00	0.009	0.922
MSTRG.6324	*CMTR2*	CAP methyltransferase 2	3.73	2.67	−1.53	0.010	0.922
MSTRG.12043	*NYAP2*	Neuronal tyrosine-phosphorylated phosphoinositide3-kinase adaptor 2	1.1	3.04	1.31	0.014	0.922
MSTRG.10771	*CCL19*	C-C motif chemokine ligand 19	5.33	10.22	1.05	0.017	0.922
MSTRG.19058	*PRR15*	Proline rich 15	3.57	2.02	−1.05	0.018	0.922
MSTRG.16341	*ASS1*	Argininosuccinate synthase 1	4.96	13.22	1.32	0.026	0.922
MSTRG.6232	*CES2*	Carboxylesterase 2	21.13	13.63	−1.02	0.027	0.922
MSTRG.18314	*CCND2*	Cyclin D2	6.25	<0.001	−1.99	0.029	0.922
MSTRG.22347	*DUOXA2*	Dual oxidase maturation factor 2	1.19	9.26	1.77	0.033	0.922
MSTRG.22348	*DUOX2*	Dual oxidase 2	1.7	12.07	1.63	0.038	0.922
MSTRG.5089	*PGAM1*	Phosphoglycerate mutase 1	17.72	34.79	2.36	0.041	0.922
MSTRG.3081	*CCL8*	Phosphoglycerate mutase 1	1.79	6.12	1.09	0.043	0.922
MSTRG.21722	*CRAMP1*	CAMP-regulated antimicrobial peptide 1	2.77	2.05	−1.02	0.044	0.922
MSTRG.24683	*SOCS3*	Suppressor of cytokine signaling 3	2.32	7.51	1.06	0.046	0.922
MSTRG.16036	*RBM15*	Suppressor of cytokine signaling 3	4.78	2.46	−1.39	0.046	0.922
MSTRG.4224	*ISG20*	Interferon-stimulated exonuclease gene 20	2.47	7.96	1.14	0.049	0.922

Note: CON: non-weaned group; EW: early-weaned group (weaned on day 21).

## Data Availability

All results data analyzed during this study are included in this published article and its Supplementary Information Files. Individual-level raw sequence data have been submitted to the NCBI Short Read Archive under accession code SRP567385.

## Supplementary Materials

The following supporting information can be downloaded at: https://www.mdpi.com/article/10.3390/ani15142135/s1. Table S1: The sequencing data mapping statistics and reference genome comparison; Table S2: List and description of DEGs (EW49 vs CON49); List and description of DEGs (EW26vs CON26).
