# Supplementary material for: Impacts of Early Weaning on Lamb Gut Health and Immune Function: Short-Term and Long-Term Effects
Source: Animals (Basel). 2025 Jul 18;15(14):2135. doi: 10.3390/ani15142135 (PMC12291703; doi:10.3390/ani15142135)
Supplement: Supplementary file 1 [file animals-15-02135-s001.zip › Table S1.pdf]

Table S1. The sequencing data mapping statistics and reference genome comparison

Items	Group				SEM
	CON26	EW26	CON49	EW49	
Valid reads	51,500,963	57,005,679	44,828,438	44,614,390	8,747,798
Mapped reads（%）	87.70%	87.86%	89.19%	89.47%	0.92%
Unique mapped reads（%）	55.30%	55.54%	66.08%	66.92%	5.70%
Multi mapped reads（%）	32.39%	32.32%	23.11%	22.56%	4.89%
PE mapped reads（%）	80.31%	80.64%	83.06%	83.44%	1.56%

Note: CON26 and CON49 refer to unweaned control lambs sampled at 26 and 49 days of age, corresponding to 5 and 28 days post-weaning in the weaned groups. EW26 and EW49 refer to lambs weaned at 21 days of age and sampled at 5 and 28 days post-weaning, respectively.
